# Are Parents of Preschool Children Inclined to Give Consent for Participation in Nutritional Clinical Trials?

**DOI:** 10.1371/journal.pone.0163502

**Published:** 2016-10-12

**Authors:** Somashekhar Marutirao Nimbalkar, Dipen Vasudev Patel, Ajay Gajanan Phatak

**Affiliations:** 1 Department of Pediatrics, Shree Krishna Hospital, Pramukhswami Medical College, Karamsad, Anand, Gujarat, India; 2 Central Research Services, Charutar Arogya Mandal, Karamsad, Anand, Gujarat, India; Old Dominion University, UNITED STATES

## Abstract

**Objective:**

Micronutrient deficiencies can lead to anemia, growth restriction, and poor motor and cognitive development. A clinical trial was planned to assess the impact of nutritional supplementation on cognitive measures in preschool children. Conducting clinical trials in children is difficult due to underlying laws, hesitation of the research community, and difficult enrollment. We carried out a questionnaire-based feasibility survey to assess the interest of parents towards participation in such a nutrition-based study.

**Methods:**

After approval from the Institutional Human Research Ethics Committee, the principals of four kindergarten schools at Vallabh Vidyanagar, Anand, Gujarat, India consented to participate. Children at the participating schools were distributed a consent form and pre-tested questionnaire, to be taken home for parents to sign, fill and return.

**Results:**

Out of a total of 1049 consent forms and questionnaires distributed, 602 (57.39%) signed and filled forms were returned. Despite fair awareness regarding the need of research, parents’ willingness to involve their children in a 6 month duration research study, not requiring invasive measures like blood pricks, was 180 (29.9%). Nearly half (250, 41.5%) did not respond and more than a quarter (172, 28.6%) declined participation on behalf of their children.

**Conclusion:**

The interest level of a pre-school child's parents for participation of the child in a nutrition intervention study evaluating cognitive measures like memory is low. Understanding the study population’s motivating and inhibiting factors leading to decreased participation in clinical trials is necessary to facilitate the creation of a pertinent evidence base.

## Introduction

The last century has seen unprecedented progress in the field of medicine. Apart from the growing economy, evolution of modern medicine through systematic and pertinent research is a key factor leading to this improvement. It may be argued that this has benefited various age groups differently. A stark gap exists between adult and childhood research [1]. Use of off-label and/or unlicensed drugs in hospitalized children is rampant, albeit limited evidence from settings outside of developed countries [2]. This situation is due to the fact that most clinical trials are conducted in adults and results from these studies are extrapolated to children. Children have a different natural history and disease pattern; this can be coupled with the fact that they may metabolize medicines differently than adults. This may result in sub-optimal treatment, along with adverse drug reactions and toxicity, resulting in undesired outcomes [3]. This exposure to untested molecules in children is due to a hesitation of the research community to involve children in clinical trials. The risk of funding a study involving children may entail many hurdles, such as 1) ethical committees disallowing studies in view of high perceived risk or minimal benefit, 2) inherent vulnerabilities requiring additional protection as research participants, 3) failure to obtain consent and/or assent from participants, 4) inability to recruit healthy pediatric volunteers, 5) a developing physical, social, cognitive, and emotional milieu, 6) preparation of appropriate formulations, 7) limited financial returns, etc [3–6]. Harry Shirkey referred to children as “Therapeutic Orphans” about 35 years ago and the situation has yet not changed [6].

There have been tremendous benefits from trials conducted in children. The childhood 5-year survival rate of acute lymphoblastic leukemia (ALL) improved from 25% to more than 70% due to multi-center trials. Recognizing the need of clinical trials in children, many research bodies and professional organizations have stressed the importance of assessing health-care interventions that are used in children [3]. In United States of America (USA), the National Institutes of Health (NIH) mandated that children be included in all human subject research that is supported by NIH unless specific reasons exist to for exclusion. There has been a steady evolution of laws and regulations in the USA and the European Union to promote clinical trials in children, with some extra incentives like extension of patent period and data protection for new indications, etc. These and other initiatives led to some improvement in clinical trials performed on children over a period [3]. The real challenges in clinical trials in children are difficulty in recruitment and follow-up of children leading to delays, cost rise, and failure to complete a study. As children below 9 years of age are not expected to give assent, the informed consent process leading to recruitment is mostly undertaken by the parents or legal guardians. It is necessary to have an insight into the thought process of parents that allows them to take appropriate decisions on behalf of the children [3].

The disease burden is high in low- and middle-income countries (LMICs) and it is imperative to generate evidence pertinent to this population. Unfortunately, it is more challenging to conduct clinical trials in children of LMICs due to various factors like logistics, fund allocation, risk of exploitation etc [7].

India has an estimated one-third of the world’s wasted children. In Bihar, 27.1% of children under the age of five were wasted and a larger number are expected to have micronutrient deficiencies [8]. Micronutrients play an important role in learning and cognitive functions, microbial virulence, cellular and humoral immune response, work capacity, etc. Micronutrient deficiencies can lead to anemia, growth restriction and poor motor and cognitive development, and can impact long term health and productivity [9]. A randomized controlled trial (RCT) was planned to assess the impact of nutritional supplementation on cognitive measures in preschool children. The logistics and resources required for the trial were immense and hence we conducted a pre-trial survey to assess the interest of the parents in the geographic area where the trial was intended to be conducted.

## Methods

The Institutional Human Research Ethics Committee of the HM Patel Center for Medical Care and Education, Karamsad, Anand, Gujarat, India approved the study.

### Study design

A questionnaire-based survey.

### Study site

School principals of four kindergarten (KG) schools situated in Vallabh Vidyanagar, in Anand District, Gujarat, India were approached and all four approved the study. Middle class parents of this area generally favor these schools, and this was the basis for school selection.

### Questionnaire

The investigators developed a questionnaire in English and then translated it into Gujarati. It was translated back to English as well, to ensure correct translation. The translations were performed by independent experts who have expertise in both languages. The questionnaire was then pre-tested on a sample of 10 parents (either mother or father) of children for clarity, completeness, acceptability and ease of answering. After making a few changes in the language and grammar of the questions, the questionnaires were administered again to 25 parents of preschool children by the investigators as a pilot. These participant parents were from schools in the same area having a similar socio-demographic mix. The 13 questions with Yes/No options in the questionnaire were then finalized. The first three questions (a to c) were related to science, technology and research; the rest of the questions (1 to 10) were related to awareness with regard to nutrition and willingness to consent for their children to participate in a nutrition related clinical trial.

### Study procedure

A hard copy of the consent form and survey questionnaire (in Gujarati) was distributed to children studying in junior KG and senior KG sections of these four schools.

Children at the participating schools were distributed a consent form and pre-tested questionnaire, to be taken home for parents to sign, fill and return.

The teachers collected these forms. A response from parents was requested in five days, but forms were also collected after five days as well, by the social workers and investigators who made repeated visits on three different occasions to collect the forms.

### Statistical analysis

The data were entered into a Microsoft Excel spreadsheet (S1 File) and descriptive analysis and correlation tests were performed using Pearson chi-square test.

## Results

A total of 1049 consent forms and questionnaires were distributed to the school children. Of these, 602 (57.39%) completed forms were collected by the teachers and returned to the investigators. Interest of the parents regarding participation of their children in nutrition-based research is demonstrated in Table 1 General awareness regarding the need of research was good among the participants. We had 180 (29.9%) parents who were willing to involve their children in nutrition-based research for the duration of 6 months without blood pricks, with complete understanding that randomization would be used to designate the intervention and control groups. Questions 8, 9, and 10 showed many non-responses (35–40%).

**Table 1:** Interest of parents regarding participation of their children in nutrition-based research (n = 602).

Question	Check marked “Yes” (%)	Check marked“No” (%)	Kept blank (%)
a) Do you think that humans have made progress in terms of newer technologies in our life? E.g., Mobiles, Television, Safer Cars, Bigger and Safer Buildings, etc.	574(95.3)	26(4.3)	2(0.3)
b) Do you think that medicines have become safer and more powerful and research has contributed to decreasing diseases in humans? E.g., Smallpox, Polio, Some cancers, etc	565(93.9)	33(5.5)	4(0.7)
c) Do you know that many medicines are available to adults first and are used in children many years after they are discovered because research in medications is initially conducted in adults and later on in children?	424(70.4)	161(26.7)	17(2.8)
1) Are you aware that under nutrition in children in India is more than that in poorer African nations and India houses the most under nourished children in the world?	385(64.0)	208(34.6)	9(1.5)
2) Do you know that deficiencies of vitamins and minerals are present not only in poor families but may also be present in the richer families due to various reasons such as, unbalanced diet, etc?	523(86.9)	72(12.0)	7(1.2)
3) Are you aware that good nutrition in young children is closely related to growth and development of brain in growing years?	569(94.5)	28(4.7)	5(0.8)
4) Would you be willing to further the cause of science by supporting and participating in nutrition related research?	394(65.4)	194(32.2)	14(2.3)
5) Will you be willing to let your child participate in a clinical study which proposes to evaluate the impact of a nutritional beverage powder on memory?	269(44.7)	320(53.2)	13(2.2)
6) Will you be willing to allow your child to participate in a clinical study in which he/she may be asked to consume a health drink daily for a continuous period of 6 months?	227(37.7)	358(59.5)	17(2.8)
7) Will you allow your child to participate in 6 months nutrition related research?	249(41.4)	327(54.3)	26(4.3)
8) Will you allow your child to participate if there is NO blood drawn during the study?	188(31.2)	190(31.6)	224(37.2)
9) Would you be willing for your child to participate in activity camps during holidays?	252(41.9)	137(22.8)	213(35.4)
10) The way nutrition studies are designed is that the participants are divided into two groups for example an active ingredient group and energy matched group with less or no active ingredients. If you consent for such a study the chance that you may enter either group is 50% and is not known to the study team. The product group allocation is found only when analysis is complete. Would you be willing to participate knowing that your child may not receive the test product which may have more benefits?	180(29.9)	172(28.6)	250(41.5)

Knowledge with regard to science, technology, and research, as well as general awareness of nutrition-related issues, did not contribute much towards increasing/decreasing participation in the nutrition-related clinical trial (Table 2).

**Table 2:** Association of parents’ knowledge and awareness with willingness to participate.

Knowledge/Awareness	Answer	Question 10: Willingness to participate		p value
		No (%)	Yes (%)	
a: Technical Progress	No	8 (36.4%)	14 (63.6%)	0.237
	Yes	162 (49.4%)	166 (50.6%)	
b: Medicine Safety	No	13 (68.4%)	6 (31.6%)	0.075
	Yes	156 (47.4%)	173 (52.6%)	
c: Transition of Medicines from Adults to Children	No	54 (58.1%)	39 (41.9%)	0.026
	Yes	111 (44.6%)	138 (55.4%)	
1: India has most undernourished children	No	64 (53.8%)	55 (46.2%)	0.16
	Yes	105 (45.9%)	124 (54.1%)	
2: Vitamin and Mineral deficiency in all socioeconomic strata.	No	25 (61.0%)	16 (39.0%)	0.08
	Yes	142 (46.4%)	164 (53.6%)	
3: Nutrition related to growth and development	No	10 (58.8%)	7 (41.2%)	0.379
	Yes	159 (47.9%)	173 (52.1%)	
4: Willing to further cause of science	No	50 (80.6%)	12 (19.4%)	<0.001
	Yes	118 (41.4%)	167 (58.6%)	

## Discussion

The current study was conducted as a stand-alone survey about participation in clinical trials of preschool children. We were not able to find similar studies in healthy children of India; we therefore believe that the results of this effort will be valuable for all clinical researchers working with children. The parents did not have an opportunity to consider a specific trial and then consent for it. It is possible that, if they were given a specific opportunity to review trial parameters, the parents would have responded with clearer answers for the questions that they were hesitant to answer. However, the parent's being able to give a generalized opinion on participation in randomized research is nevertheless beneficial.

The investigators consider the participation rate of 57.4% as quite high, taking into consideration that all of the participants were parents with healthy, school-going children. A study by Vanhelst, et al. [10] demonstrated only a 32% participation rate among healthy children as compared to 93% among ambulatory sick children. This study, conducted in France, used a questionnaire that was developed after a focus group discussion. It included several factors such as demographic and social information, motivational factors, and an exploration into factors that could improve study participation. A similar study in sick children had a participation rate of 59% [11]. In another survey carried out in parents, whose children were approached for participation in randomized clinical trials during the preoperative period (for 15 anesthesia and three surgical studies), there was an 88% participation rate. In this study the predictors of consent for participation in a randomized trial were low perceived risks, parental understanding, reading the informed consent document fully, the characteristics of the consent document, perceived importance of the research, and perceived benefits [12]. However, these studies have been carried out in parents who already had children completing clinical trials, unlike ours which was a pre-trial survey. There was a drop of almost 10% in participation rate when the parents were given a scenario wherein their child may not get into the intervention group. This is possibly because the idea of getting randomized to a placebo group or to a group receiving ineffective treatments, along with potential side effects and the inconvenience of participation was considered a risk by the parents [13]. Parents perceived benefits in participation when they understood that they may get better care for their child, newer treatments and access to better healthcare professionals and health information. The child’s health status also altered participation in clinical trials, with parents of sick children more likely to consent [13]. The current study, being a pre-trial survey regarding participation in a clinical nutritional study, was purely exploratory in nature and did not have serious illnesses to contend with, unlike most of the studies that explore participation in clinical trials. Informed consent for participation in clinical trials does not seek to safeguard a child’s autonomy, but it is maintained mainly to preserve wellbeing. Parental consent is not expected to offer additional protection as current regulatory scenarios will take care of safety issues. Rather, parental consent seeks to maintain social recognition of parents and in studies where active participation to maintain records may be required, this becomes even more important. Parents will have varying degrees of trust in a particular study’s research team and this factor influences the outcome of informed consent [14]. They are more likely to rely on the treating physician’s recommendations and/or expertise when making a decision, especially if their child is sick. A parent with a sicker child, who has an illness with possible mortality in the near future, will experience great distress in making decisions. In less serious chronic diseases, such as childhood asthma, many parents allowed participation, due to their interest regarding the disease. The daily management of a particular illness, its quality of life lead, and the burden imposed by trial participation become major deciding factors [14]. However, parents of a healthy child, who will avail no direct benefit from the study, may be inclined to make a decision based solely on the balance between the burden imposed by the trial and the perceived benefits of participation. Trial participation rates are, therefore, more likely to be lower when there is no perceived or actual illness due to the expected burden of the study.

While 32.2% did not want to participate in research, the figure increased by more than 65% to 53.2% when it came to allowing their own child to participate in a research study. The scenarios in the questionnaires (Questions 4 and 5) were related, but not identical. The willingness to participate further declined, with 59.5% of the parents refusing to give consent for their children to participate in a nutrition-related study of longer duration. A qualitative study in sickle cell disease patients and parents, performed in Philadelphia, reported that caregivers are more likely to consider potential harm when making the decision, while children are more likely to consider the benefit. Potential benefit, manageable study demands, and trust in clinical staff were of secondary importance and hence a suggestion is made that potential harm needs to be addressed first, followed by benefits and manageable demands. This assumes importance since the safety issues in a large majority of studies increase study burden, for both participants and investigators [15]. The parents may perceive more harm from research for their children as compared to themselves, and this may explain the steep 65% rise in considering no participation in a randomized trial. A separate study, related not to research participation, but to postponement of clinical care, showed that mothers are more than twice as likely to postpone care for themselves, as compared to their children, due to cost considerations [16]. While this study is in a different context, it is from the same geographic location and demonstrates that parents are more likely to consider risk and/or poor quality care for their child's health, as compared to their own health. Here the concept of autonomy and informed consent assumes importance. Children are more likely to participate in clinical trials, when asked, as compared to their parents due to different evaluation of perceived risks and benefits. However, children desirous of participation (especially adolescents), can only do it if their parents give informed consent.

Questions 8, 9 and 10 were answered by fewer than 35% of the parents, as compared to the previous seven questions that were answered by more than 95% of parents. It is a gratifying number to the researcher, as it indicates that the parents required more information before making the decision. The enthusiasm may be misplaced, since studies conducted elsewhere have had mixed results. In a multi-center study conducted across the US, parents with high socio-economic status were more likely to decline participation of their children as compared to those less educated or from a lower socioeconomic status. The parents who consented displayed higher levels of trust and, displayed more altruism. The manner in which consent was sought, whether the parents felt comfortable with the researcher also affected participation. Perceived risks and financial incentives did not play any role in decision-making. This particular study compared consenters and non-consenters and utilized a detailed questionnaire, of about 60 questions, that was filled out after a study on vesico-ureteral reflux was completed. The amount of participation in the survey was similar between the two groups [17]. We correlated high level of awareness and knowledge for science, technology, and research to high education level, and we found no difference of it towards participation in clinical trial. By contrast a study from University of Michigan Children’s Hospital, on parental permission for allowing their child to participate in a clinical anesthesiology or a surgical study, demonstrated that education level lower than high school increased the consent rate [12]. However, the study’s questionnaire differentiates knowledge and awareness into just two categories (“Yes” and “No”) in the present study. It is likely that the "yes" category could include parents with very little to a great deal of knowledge/awareness, thereby masking effects on willingness to participate along this continuum. Thus, the association between knowledge/awareness and decision to participate in a trial may not be accurate.

Social, cultural, educational, and economic conditions vary across countries and these assume a great importance in decision-making. A small survey, conducted in the capital city of India, among adults demonstrated a positive response to research, with 58.9% of 175 respondents being open to participation in research. They perceived participation in research as voluntary (85.3%) and agreed that research benefits society (94.1%), which was similar to the responses in our study. Altruism was one of the main reasons (62.5%) for the increased willingness to participate in this study’s Indian adult population, as compared to 39% (USA) and 35% (non-USA countries) [18]. Willingness to contribute towards science through participating was the motivating factor in the current study. An older meta-analysis of qualitative studies from India displayed altruism, personal health benefits, trust of physicians, detailed knowledge about trials, source of extra income, and methods for motivating participants as the themes that favored participation. The same study displayed mistrust for trial organizations, psychological reasons, concerns about efficacy and safety of trials, loss of confidentiality, trial burden, dependency issues and language as factors restricting participation. India is a very large country with varying cultures across its population. Therefore, the reasons behind participation or declining consent may need to be studied in various areas and among different populations, in order to have a more unbiased view on this issue. We also need to study this at regular intervals, as perception may change with time due to varying influencing processes such as main stream media, economy, social media, etc.

The authors therefore conclude that awareness regarding the need of research in the day-to-day life of participants was fair. In general, there was a moderate level of interest in the study but the parents’ keenness to participate was guarded. The generalizability of the study results is limited; since the questionnaire was not assessed in continuum. This is coupled with fact that it was a single center study with a convenient sampling frame. Further research, including more qualitative aspects, is warranted in order to comprehensively understand the perceptions among parents regarding clinical trials in the study population.

## Supporting Information

S1 File Data of the study. (XLS)
